# Supplementary material for: Human CD1c-autoreactive T-cells recognise Mycobacterium tuberculosis-infected antigen-presenting cells and display cytotoxic effector programmes
Source: eLife. 2026 Oct 5;15:RP110341. doi: 10.7554/eLife.110341 (PMC13638199; doi:10.7554/eLife.110341)
Supplement: Supplementary file 2 [file elife-110341-supp1.docx]

**Table S1. Primer sequences used for targeted single-cell TCR sequencing.**

Sequences are shown for the oligo-dT primer, universal forward and reverse primers, targeted forward primer containing the 8-bp well barcode, and reverse primers specific for TRAC, TRBC, TRGC and TRDC constant regions.

| Oligo-dT | GGAATTCGACCGAGTACACATAAGGT30VN |
| --- | --- |
| Universal Forward | ACGTCACTCACTCGTAGCCTAAGCAGTGGTATCAACGCAGAGT |
| Universal Reverse | GGAATTCGACCGAGTACACATAAGG |
| Targeted Forward | 8 bp well barcode-ACGTCACTCACTCGTAGCCT |
| Targeted Reverse TRAC | GAGTCTCTCAGCTGGTACACGG |
| Targeted Reverse TRBC | AGTGTGGCCTTTTGGGTGTG |
| Targeted Reverse TRGC | GGTATGTTCCAGCCTTCTGGAG |
| Targeted Reverse TRDC | GGGAGAGATGACAATAGCAGGATC |
