# Supplementary material for: Human CD1c-autoreactive T-cells recognise Mycobacterium tuberculosis-infected antigen-presenting cells and display cytotoxic effector programmes
Source: eLife. 2026 Oct 5;15:RP110341. doi: 10.7554/eLife.110341 (PMC13638199; doi:10.7554/eLife.110341)
Supplement: Peer review history

# Editor report: eLife Assessment

**DOI:** 10.7554/eLife.110341.3.sa0

**Authors:** Vinay Kumar Nandicoori

**Affiliations:** National Institute of Immunology India

**Keywords:** Important, Solid

The study investigates how CD1c-restricted T cells respond to Mtb-infected APCs, leading to increased cytokine production and cytotoxic activity that may help control Mtb infection. The work is **important** and will interest researchers in the field. The supporting evidence is **solid**.

# Referee report: Reviewer #1 (Public review)

**DOI:** 10.7554/eLife.110341.3.sa1

Summary:

T cells that recognize lipids-CD1c are frequent in circulation; however, their role in infection is unclear. This study aims to understand how Mtb infection can shape the responses of CD1c-specific T cells. CD1c is expressed in MTB granuloma, but in lower amount than in nearby inflamed tissue. Mtb infection downregulates the expression of CD1c on monocyte derived DCs. Single cell RNA sequencing revealed the cytotoxic program inherent to the lipids-CD1c-specific T cells. Using an in vitro APC system where CD1c expression remains intact upon Mtb infection, the authors suggest that these T cells react better to Mtb-infected than uninfected Cd1c-expressing APC and reduce Mtb burden in infected cells. Therefore, Cd1c downregulation could be an immune evasion strategy used by Mtb.

Strengths:

This study asks an important question. The single cell transcription analysis suggests the inherent cytotoxic program of lipid-CD1c cells and provides insights into their phenotypic and potential functional profiles. Function experiments suggest that these autoreactive T cells can react to Mtb infection, adding to the paradigm of infection control by these non-conventional T cell population. In summary, this study reports potential role of lipid-CD1c specific T cells in Mtb infection., and the evidence overall supports the conclusions.

Weaknesses:

(1) The study engineered THP-1 cells that lacked endogenous beta2 microglobulin (beta2m) and stably expressed chimeric CD1c-beta2m (CD1c-THP-1 cells). T cells isolated from healthy donors showed stimulation when mixed with CD1c-THP-1, suggesting the presence of CD1c reactive T cells in healthy individuals (Fig. 1). It appears that antibody staining profiles for different markers are not from the same experiment (Fig. 1). It is better to show all the representative antibody staining from a single experiment. And a quantification of MFI values from at least three independent experiments should be shown with statistics. Further, in each of the representative antibody profile, unstained/isotype control should be incorporated. The characterization of beta2m KO cells - using DNA sequencing and western blotting - should be shown in supplementary.

(2) Next, using immunohistochemistry, the study suggests expression of CD1c in lung biopsy from TB patients, and that CD1c expression is reduced on primary MoDCs upon Mtb infection (Fig. 2).

(3) To assess CD1c-reactive T cell cytotoxicity in Mtb infection, the authors generated CD1c-T cell lines (from T cells from healthy individuals) using two different approaches. In one, CD1c-specific T cells were expanded upon mixing with CD1c-THP-1 cells, were sorted using CD1c-endo tetramer, and expanded. In another approach, CD1c-specifc T cells were sorted (using CD1c-endo dextramer) directly from blood-derived T cells and were expanded. The line obtained using one of the approaches to assess cytotoxicity towards CD1c-THP-1 cells, uninfected or infected with Mtb. It is seen that CD1c-T cells exert cytotoxicity towards infected, and not towards uninfected, CD1c-THP-1 cells (Figs. 3,4).

The authors should clearly indicate the T line, obtained from which one of the two approaches, was used for the cytotoxicity assay, and what was the result with the line obtained from other approach. Fig. S4 shows the sorting strategy and final profile of the T line obtained after CD1c-endo tetramer-based sorting and expansion. This figure is mistakenly referred as supplementary fig. 3 in the caption of Fig. 3; should be corrected. Further, to complete the Fig. S4, the intermediate step of sorting scheme, which shows negative and positive fraction as defined by streptamer binding, should be included. And Y-axis of left-most lower-most panel (no tetramer) should be labelled. The Profile of the T line shows three distinct population: tetramer-low, tetramer-intermediate, and tetramer-high. The authors should comment on this, particularly on the tetramerlow population that can be CD1c-negative cells that have non-specific weak binding to the tetramer.

The profile of the T line obtained from the other approach should also be shown, and the experiments that used those should be clearly indicated.

(4) To assess whether the elevated response of CD1c-T cells is mediated by the T cell receptor (TCR) that they harbour, the authors performed single T cell sequencing for TCR. They could identify 11 αβ pairs, two of them (named as EM1 and EM2) constituted 10 out of the 11 αβ pairs. It is surprising that such a shallow sampling (despite of the filtering mentioned in Fig. S9) such a high degree of clonal expansion and yielded two context-relevant TCR sequences. These TCRs were indeed DC1c specific, as indicated by their ability to upregulate CD69 upon engagement with CD1c (Fig. 5C). However, they respond very weakly to CD1c-THP-1 cells - the difference (THP-1 KO versus CD1c-THP-1 cells) is small for EM1, and the upregulation of CD69 per se is very weak (within the noise range) for EM2 (Fig. 5D). Nonetheless, the difference shown is statistically significant for both EM1 and EM2.

(5) The study further indicate that the CD1c-specific T cells are enriched for the marker associated with their cytotoxic functions, and they perform slightly better in controlling Mtb growth in culture.

The authors are suggested to carefully go through the manuscript a couple of times so that supplementary figures are referred appropriately and accurately.

# Referee report: Reviewer #2 (Public review)

**DOI:** 10.7554/eLife.110341.3.sa2

Summary:

The study by Milton et al titled "Human CD1c-autoreactive T cells recognise *Mycobacterium tuberculosis*-infected antigen-presenting cells and display cytotoxic effector programmes" characterises CD1c-restricted autoreactive T cells and their potential role in controlling Mtb infection. The authors develop a well-controlled system to assay for the functioning/activation of autoreactive T cells. They report the presence of CD1c-restricted autoreactive T cells in the circulating blood of healthy donors. They show that these T cells respond to CD1c and get activated even in the absence of any exogenous antigen. They next show that CD1c, along with CD1a and b, are typically downregulated on APCs during Mtb infection. These autoreactive T cells are cytotoxic, indicating they respond to Mtb treatment and/or to changes in the T cell ratio. The autoreactive T cells could effectively lyse Mtb-infected or PAMP-stimulated CD1c+APCs. Next, using TCR sequencing, they show that T cell responses were mediated by specific TCR clones with common sequence features. They show that these autoreactive T cells could curtail Mtb growth as measured by luminescence. Finally, using scRNAseq, they selectively identify the CD1c-reactive T cell pool and detect enrichment of typical effector memory CD4 and CD8 cells expressing cytolytic markers such as Granzyme, granulolysin, etc. The lung biopsy staining, along with the other data presented here, suggests that while CD1c-restricted T cells could have potential anti-bacterial roles, Mtb downregulation effectively shuts down this mechanism for TB control.

Strengths:

The study is designed well and has developed many exciting tools to generate specific information.

Weaknesses:

The revised manuscript addresses many concerns, but one section remains weak. The efficiency of these CD1c-restricted T cells in controlling TB remains very limited. The only result that addresses the bacterial control through this mechanism is Fig. 6C, which shows a very modest impact. Even THP1-KO cells show a decline in CFU when cultured with autoreactive CD1c-autoreactive T cells, and the further dip in THP1 CD1c cells is very minimal.

Another issue left unaddressed is the cytolytic response on Mtb-infected cells. How efficient are lytic responses in controlling Mtb infection? Usually, bacteria can emerge from lysed cells and divide extracellularly. How would one show this mechanism in vivo?

# Referee report: Reviewer #3 (Public review)

**DOI:** 10.7554/eLife.110341.3.sa3

The authors have addressed most concerns from the initial review, significantly enhancing the manuscript. The expanded characterisation of the engineered THP1-CD1c system provides strong evidence that the observed T-cell responses are unlikely to result from residual conventional MHC recognition. The specificity of the CD1c-autoreactive T-cell lines is further confirmed by multimer staining, their inactivity against THP1-KO cells, and TCR-transfer experiments.

The revised data support the main conclusion that human CD1c-autoreactive T-cells recognise Mtb-infected cells that express Cd1c and exhibit cytotoxic effector functions. The difference between TCR-dependent recognition, shown by EM1 and EM2, and cytotoxicity, demonstrated by the original T-cell lines, is now more clearly presented.

Several limitations remain. The specific CD1c-associated lipid signalling molecule that enhances recognition of Mtb-infected cells has yet to be identified. Additionally, the bacterial luminescence assay lacks validation against CFU counts and cannot differentiate between intracellular and extracellular bacteria. The single-cell RNA sequencing was conducted with only two donors, and the lung immunohistochemistry remains qualitative without comparison to healthy or non-TB inflammatory tissues. These constraints limit detailed mechanistic insights and broader applicability, but they are appropriately reflected in the manuscript.

Overall, this is an important study that advances understanding of human CD1c-autoreactive T-cells in the context of mycobacterial infection. The evidence supporting the principal conclusions is solid, provided that altered CD1c-associated lipid presentation remains as a mechanistic hypothesis and the reduction in Mtb luminescence is not taken as direct evidence of selective intracellular bacterial killing.

# Author comment: Author response

**DOI:** 10.7554/eLife.110341.3.sa4

**Authors:** Matthew Milton, Sahar Farag, Diana J Garay-Baquero, Jennie Gullick, Kinga Niedobecka, Daniel Burns, Rita Szoke-Kovacs, Patrick Trimby-Smith, Alex Look, Richard Stopforth, Marco Lepore, David K Cole, Laura Denney, Andrew White, Sally Sharpe, Alasdair Leslie, Andres Vallejo, Liku B Tezera, Paul T Elkington, Salah Mansour

**Affiliations:** University of Southampton Southampton United Kingdom; University of Southampton Southampton United Kingdom; University of Southampton Southampton United Kingdom; University of Southampton Southampton United Kingdom; University of Southampton Southampton United Kingdom; University of Southampton Southampton United Kingdom; Immunocore Abingdon United Kingdom; University of Oxford Oxford United Kingdom; University of Southampton Southampton United Kingdom; University of Cambridge Cambridge United Kingdom; Immunocore Abingdon United Kingdom; Immunocore Abingdon United Kingdom; University of Southampton Southampton United Kingdom; UK Health Security Agency Porton Down United Kingdom; UK Health Security Agency Porton down United Kingdom; Africa Health Research Institute Durban South Africa; University of Southampton Southampton United Kingdom; University of Southampton Southampton United Kingdom; University of Southampton Southampton United Kingdom; University of Southampton Southampton United Kingdom

The following is the authors’ response to the original reviews.

We have carefully considered all comments and have revised the manuscript to address the key points raised. We have also updated the author list to include Kinga Niedobecka, who performed the additional flow cytometric validation of the engineered THP1 cell lines included in the revised manuscript. In particular, we have strengthened the validation of the THP1-CD1c system, clarified and better signposted the characterisation of CD1c-autoreactive T-cells using existing data, and refined the explanation of the mechanisms underlying enhanced responses to Mtb-infected cells. Some of the suggestions represent significant additional experimental work beyond the scope of this manuscript, and in these instances we have amended the text to clarify interpretation and limitations.

**eLife Assessment**

The study investigates how CD1c-restricted T cells respond to Mtb-infected APCs, leading to increased cytokine production and cytotoxic activity that may help control Mtb infection. While the work is important and will interest researchers in the field, the supporting evidence is incomplete and could be strengthened by additional experiments. Experiments would: (i) evaluate THP1-CD1c cells to determine whether MHC surface expression is reduced or entirely abolished, (ii) enhance confidence in the purity of the CD1c-specific T cell population isolated from blood, and (iii) suggest what additional signal THP1-CD1c cells treated with Mtb express that is absent from the untreated cells.

(i) evaluate THP1-CD1c cells to determine whether MHC surface expression is reduced or entirely abolished

We thank the Editor for highlighting this important point. We agree that it is essential to establish whether conventional MHC-mediated antigen presentation could contribute to the observed T-cell responses. To address this directly, we repeated and extended our flow cytometric validation of the engineered THP1 system. These data are now presented in an expanded Fig. 1A and include assessment of CD1c, classical MHC class I, MHC class II, β2m, CD1b and HLA-E across WT THP1, THP1-KO and THP1-CD1c cells. Our THP1-KO system is based on CRISPR-mediated knockout of both β2microglobulin (β2m) and the Class II transactivator (CIITA). Loss of β2m removes surface expression of β2m-dependent molecules, including classical MHC class I and endogenous CD1 proteins, while CIITA knockout prevents MHC class II expression. In this new analysis, WT THP1 cells expressed β2m and classical MHC class I, with low detectable MHC class II and HLA-E. In contrast, THP1-KO cells lacked detectable β2m, MHC class I, MHC class II, HLA-E, CD1b and CD1c. Importantly, THP1-CD1c cells retained robust CD1c expression through the CD1c-β2m fusion construct, while MHC class I, MHC class II, CD1b and HLA-E remained undetectable by flow cytometry.

These extended validation data support the conclusion that residual MHC expression does not account for the observed T-cell responses, which are instead dependent on CD1c expression. We have revised the relevant section of the Results to incorporate these data and to clarify that the engineered THP1-CD1c APC system provides robust CD1c expression in the absence of detectable surface MHC-I or MHC-II (revised manuscript, page 5-6, lines 111-122; Fig. 1A and Fig. 1 legend).

(ii) enhance confidence in the purity of the CD1c-specific T-cell population isolated from blood

We agree that confidence in the specificity and purity of the CD1cautoreactive T-cell populations is essential. The relevant data were included in the original manuscript, but we recognise that they were not signposted clearly enough. We have therefore revised the Results to describe the enrichment, sorting, post-expansion validation and functional specificity of the T-cell lines more explicitly on page 8, lines 176-191.

CD1c-autoreactive T-cell lines were generated from two independent donors using two complementary strategies. One line was generated by expansion with THP1-CD1c APCs followed by CD1c-endo tetramer-guided sorting and expansion. A second line was generated by direct enrichment using CD1c-endo streptamers, followed by CD1c-endo dextramer sorting and expansion. The gating strategy and post-sort validation are shown in Fig. S4. Importantly, after expansion, the enriched cells stained strongly with CD1c-endo tetramers, whereas unstained and irrelevant tetramer controls showed no detectable staining. In the main figure, both donor-derived lines are shown to be strongly CD1c-endo tetramer-positive, with post-expansion tetramer positivity of 97100% (Fig. 3A and 3C). Both lines were αβTCR+CD4+ and lacked detectable γδTCR or CD8 expression (Fig. 3B and 3D).

We also highlight the functional validation of specificity. Both T-cell lines were activated by THP1-CD1c APCs but not THP1-KO APCs, as assessed by CD69 and CD25 upregulation (Fig. 3E). Importantly, the specificity of these cells was further supported by TCR transfer experiments. TCRs cloned from one of the CD1c-endo tetramer-positive T-cell lines were expressed in Jurkat reporter cells and conferred CD1c-endo tetramer binding, activation in response to plate-bound CD1c-endo protein, and enhanced activation in response to Mtb-infected THP1-CD1c APCs (Fig. 5B-D, page 10, lines 225244). This provides independent confirmation that the enriched T-cell line contained CD1c-reactive TCRs capable of mediating CD1c-dependent recognition.

Together, these data support that the T-cell populations used in the functional assays are highly enriched CD1c-specific T-cell lines rather than mixed or nonspecific populations.

(iii) suggest what additional signal THP1-CD1c cells treated with Mtb express that is absent from the untreated cells.

We agree that identifying the additional signal provided by Mtb-treated THP1-CD1c cells is an important mechanistic question. We have now revised the Discussion to clarify our interpretation and to more explicitly outline the likely mechanisms (revised manuscript, page 16-17, lines 380-400).

Our data suggest that the enhanced response to Mtb-infected THP1-CD1c cells is unlikely to be explained simply by increased CD1c expression, generic APC activation, or soluble cytokine release. CD1c expression was maintained but not increased on THP1-CD1c cells after Mtb infection, and stimulation with TLR2 or TLR4 agonists did not reproduce the enhanced cytotoxicity observed after Mtb infection. In addition, Mtb-treated THP1-CD1c cells alone produced IL-8 and RANTES, but not the broader cytokine profile observed in T-cell co-cultures. Together, these data suggest that Mtb exposure provides an additional CD1c-dependent activating signal.

We now discuss that this signal is most likely an altered CD1c-presented lipid repertoire on Mtb-exposed APCs. Possible mechanisms include presentation of Mtb-derived lipids, infection-induced accumulation of host-derived stimulatory “stress lipids”, presentation of bacterial and mammalian shared lipids, or altered lipid processing and trafficking during infection. These possibilities are consistent with prior studies showing enhanced responses of autoreactive CD1-restricted T-cells to microbial stimulation and our TCR transfer experiments seemingly support a CD1c-TCR-dependent recognition mechanism. However, because we have not directly identified the lipid ligands presented by CD1c on Mtb-infected APCs, we now state this as a mechanistic hypothesis rather than a conclusion, and a key outstanding question.

We have revised the Discussion (page 16-17, lines 380-410) to make this limitation explicit. Future studies will require isolation of CD1c molecules from Mtb-infected cells and then lipidomic analysis and mass spectrometry to define the CD1c-associated lipid species.

**Reviewer #1 (Public review):**

Strengths:

(1) This study asks an important question. The single-cell transcription analysis suggests the inherent cytotoxic program of lipid-CD1c cells and provides insights into their phenotypic and potential functional profiles. Function experiments suggest that these autoreactive T-cells can react to Mtb infection, adding to the paradigm of infection control by these non-conventional T-cell populations.

We thank the reviewer for this positive assessment of the importance of the study and for recognising the value of the single-cell transcriptional analysis and functional experiments. We are pleased that the reviewer agrees that our findings provide insight into the cytotoxic effector programme of CD1c-autoreactive T-cells and their potential contribution to immune responses during Mtb infection.

Weaknesses:

(2) The study lacks sufficient rigor; conclusions may be strengthened with the incorporation of more controls, and some deeper characterization of the THP1 system and the CD1c-specific T-cells isolated from blood. Crucial conclusions are drawn from the cell mixing experiments involving the engineered THP-1 system and CD1c-lipidspecific T-cells from blood. These cells need more in-depth characterization. The expression of MHC-I/II is clearly reduced in THP1-CD1c cells. However, it is important to ensure that it is completely abolished, since a residual expression can skew the result with activation of conventional T-cells in the blood or low levels of conventional T-cells that may be present in the CD1c-tetra/multimer sorted T-cells

We agree that this is an important point and have addressed it by adding new experimental controls and by clarifying the validation of the CD1c-autoreactive T-cell lines.

First, we repeated flow cytometric validation of the existing markers and extended the panel to assess additional surface molecules across WT THP1, THP1-KO and THP1CD1c cells. The revised Fig. 1A therefore includes repeat staining for β2m, classical MHC class I, MHC class II and CD1c, together with newly added staining for HLA-E and CD1b.

The THP1-KO system is based on CRISPR-mediated knockout of both β2-microglobulin (β2m) and the Class II transactivator (CIITA). Loss of β2m removes surface expression of β2m-dependent molecules, including classical MHC class I and endogenous CD1 proteins, while CIITA knockout prevents MHC class II expression. The repeated analyses confirmed the original staining pattern, while the additional HLA-E and CD1b stains further extended validation of the system. WT THP1 cells expressed β2m and classical MHC class I, with low detectable MHC class II and HLA-E. In contrast, THP1-KO cells lacked detectable β2m, MHC class I, MHC class II, HLA-E, CD1b and CD1c.

Importantly, THP1-CD1c cells retained robust CD1c expression through the CD1c-β2m fusion construct, while MHC class I, MHC class II, HLA-E and CD1b remained undetectable by flow cytometry. We have revised the Results to describe these new validation experiments more clearly (revised manuscript, page 5-6, lines 111-122, Fig. 1A and Fig. 1 legend).

Second, we have strengthened the description of the purity and specificity of the CD1c-autoreactive T-cell lines. These lines were generated using two complementary approaches, namely expansion with THP1-CD1c APCs followed by CD1c-endo tetramer-guided sorting, and direct enrichment using CD1c-endo streptamers followed by CD1c-endo dextramer sorting and expansion. The gating strategy and post-sort validation are shown in Fig. S4. After expansion, the enriched cells stained strongly with CD1c-endo tetramers, whereas unstained and irrelevant tetramer controls showed no detectable staining. Both donor-derived lines were strongly CD1c-endo tetramer positive, with post-expansion tetramer positivity of 97 to 100%, and both were αβTCR+CD4+ with no detectable γδTCR or CD8 expression (Fig. 3A-D). Functionally, both lines responded to THP1-CD1c APCs but not parental THP1-KO APCs, as assessed by CD69 and CD25 upregulation (Fig. 3E). We have revised the Results to signpost these data more clearly (revised manuscript, page 8, lines 176–191).

Finally, TCR transfer experiments provide independent confirmation of CD1c-specific recognition. TCRs cloned from one of the CD1c-endo tetramer-positive T-cell lines conferred CD1c-endo tetramer binding and CD1c-dependent activation when expressed in Jurkat reporter cells (Fig. 5B-D, revised manuscript, page 10, lines 225244). Together, the absence of detectable MHC-I/MHC-II expression in the engineered APC system, the high CD1c-endo tetramer enrichment of the T-cell lines, the lack of activation against THP1-KO cells, and the TCR transfer experiments support the conclusion that the observed responses are driven by CD1c-dependent recognition rather than residual conventional MHC-mediated activation.

(3) Figure 2: The immunohistochemistry appears to be shown only for one biopsy; it may be worth quantifying the immunohistochemistry of all five.

We thank the reviewer for this helpful suggestion. We agree that quantitative analysis of CD1c immunohistochemistry across all biopsies would be valuable. We examined CD1c staining across all five TB lung biopsies and observed a consistent spatial pattern, with CD1c staining generally low or infrequent in central granulomatous regions and more apparent in distal inflammatory tissue and lymphoid/B-cell follicle-rich areas.

However, because these were diagnostic human biopsy samples with substantial variation in tissue size, architecture, granuloma representation and inflammatory composition, we do not think that simple bulk quantification of CD1c-positive area across biopsies would be robust or biologically interpretable. In particular, quantification would be strongly affected by whether a section captured granuloma centre, peripheral inflammatory regions, lymphoid aggregates, or uninvolved lung tissue. We have therefore retained the IHC as representative spatial evidence of CD1c expression in TB lung tissue, rather than presenting it as a quantitative comparison across anatomical compartments.

We have revised the Results to make this clearer, stating that CD1c expression was observed across the biopsies analysed but was spatially heterogeneous, with staining most apparent away from the granuloma centre and in lymphoid/inflammatory regions (revised manuscript, page 7, lines 144-151). We have also tempered the interpretation in the Discussion to avoid overstatement and now highlight systematic quantitative spatial analysis of larger tissue cohorts as an important future direction (revised manuscript, page 18, lines 427-430).

(4) The expression of CD1 molecules goes up during the differentiation of MoDC, and Mtb infection prevents or dampens the upregulation. Does Mtb infection downregulate the CD1 expression of mature DCs? Can the effect of Mtb on the expression of CD1a,b,c molecules be investigated using CD1c-expressing DCs from blood? What could be the reason THP-1 cells do not downregulate CD1 molecules upon Mtb infection, and how about the expression of CD1a and b?

We agree that the distinction between impaired CD1 upregulation during MoDC differentiation and active downregulation of CD1 expression on already differentiated CD1-expressing DCs is important.

In the revised manuscript, we have clarified that our primary cell data assess the effect of Mtb infection on differentiated MoDCs that already express CD1 molecules, rather than only examining failure of CD1 induction during differentiation. Specifically, we analysed a published RNA-sequencing dataset from differentiated human MoDCs infected with live Mtb and observed reduced expression of group 1 CD1 genes, including CD1A, CD1B and CD1C, at 48 hours after infection. We then validated this experimentally at the protein level by flow cytometry, showing reduced CD1c expression on primary MoDCs after live Mtb infection. These data support the conclusion that Mtb infection can reduce CD1 expression on CD1c-expressing primary DCs.

We agree that analysis of freshly isolated blood CD1c+ DCs would be valuable. However, these cells are rare in peripheral blood and are technically challenging to isolate in sufficient numbers for live Mtb infection assays and downstream flow cytometric or functional analysis. For this reason, we used MoDCs as a tractable primary human DC model to assess infection-induced changes in CD1 expression. We now acknowledge in the revised Discussion that validation in primary blood-derived CD1c+ DCs would be an important future direction.

We have also clarified why CD1c expression is not downregulated in the engineered THP1-CD1c system. In primary DCs, Mtb-mediated suppression of CD1c has been linked to host regulatory mechanisms, including post-transcriptional regulation by miRNAs such as miR-381-3p, which targets the 3′ UTR of endogenous CD1c transcripts. In contrast, CD1c expression in our THP1-CD1c cells is driven by a lentiviral CD1c-β2m fusion construct under a heterologous promoter and expressed from a cDNA lacking the native untranslated regions. Therefore, CD1c in this system is not expected to be regulated in the same way as endogenous CD1c in primary DCs.

This is a deliberate feature of the model. It allows us to assess CD1c-dependent T-cell responses to Mtb-infected APCs without the confounding effect of infection-induced CD1c loss. THP1-CD1c cells do not express endogenous CD1a or CD1b because the parental THP1-KO cells lack β2m-dependent endogenous CD1 surface expression, and only CD1c is reintroduced through the CD1c-β2m fusion construct. We have revised the Results and Discussion to clarify these points (revised manuscript, pages 7- 8, lines 165174 and pages 17- 18, lines 411-430).

(5) Figure 3: (F) What does the X-axis read for the no infection group? The value for MOI = 0 should be incorporated for the infected T-cell group.

We agree that the original presentation could be clearer. The uninfected condition corresponds to MOI = 0, whereas the remaining points represent THP1-CD1c APCs exposed to increasing amounts of UV-killed Mtb. We have retained the figure layout but revised the figure legend to clarify that the MOI values on the x-axis apply only to the Mtb-treated conditions, and that the no-infection/no-treatment control represents MOI = 0 (Fig. 3 legend).

(6) Figure 4: In the lysis assay, THP1-CD1c cells (uninfected and infected) incubated alone should be incorporated.

We agree that APC-only controls are essential for interpreting the lysis assay, and we apologise that this was not sufficiently clear in the original manuscript. THP1-CD1c cells cultured alone, both uninfected and Mtb-infected, were included in all assays and used to define baseline target-cell viability for each matched condition.

The data in Fig. 4 are presented as specific lysis to isolate the effect of T-cells on target cell viability. Specifically, THP1 viability in APC-only wells was used as the baseline and subtracted from the corresponding T-cell co-culture condition within the same experiment. Thus, lysis of uninfected THP1-CD1c cells was calculated relative to uninfected THP1-CD1c cells cultured alone, and lysis of Mtb-infected THP1-CD1c cells was calculated relative to Mtb-infected THP1-CD1c cells cultured alone. This presentation allows the T-cell-mediated effect to be visualised while accounting for baseline viability differences.

We have revised the Methods and Fig. 4 legend to make this calculation more explicit (revised manuscript, page 25, lines 608-614; Fig. 4 legend).

(7) A quantitative brief on the single cell TCR sequencing - including how many T-cells were sequenced and the frequency of different clone including EM1 and EM2 - should be shown.

We agree that the single-cell TCR sequencing data required clearer quantitative description. We have expanded the Results and Fig. 5 legend to include the number of single cells analysed and the frequency of the dominant clonotypes. Single CD1c-endo tetramer-positive T-cells were sorted into individual wells for targeted TCR sequencing. After filtering and manual curation, 11 single cells yielded productive paired αβ TCR sequences. The repertoire was oligoclonal, with two dominant productive clonotypes accounting for 10 of 11 paired TCRs. EM1 was detected in 6 of 11 cells and EM2 was detected in 4 of 11 cells. These data support the selection of EM1 and EM2 for TCR-transfer experiments and clarify that they were dominant clonotypes within the CD1c-endo tetramer-positive T-cell line rather than arbitrarily selected TCRs. We have revised the Results and Fig. 5 legend accordingly (revised manuscript, page 10, lines 225-235, Fig. 5 legend).

**Reviewer #1 (Recommendations for the authors):**

(8) Perform an experiment to assess activation of T-cells expressing EM1 or EM2, upon mixing with CD1c-expressing dendritic cells isolated from human blood, with and without Mtb infection.

We agree that testing EM1 and EM2 TCRs against primary dendritic cells is an important question. However, in the specific context of Mtb infection, the proposed experiment is difficult to interpret because Mtb downregulates CD1c expression on primary dendritic cells. This is supported by previous studies showing that Mtb and BCG suppress CD1c expression on DCs [1,2], and by our own data showing reduced CD1 group 1 transcript expression in Mtb-infected MoDCs and reduced CD1c protein expression on primary MoDCs following Mtb infection (Fig. 2B and 2C). Therefore, mixing EM1- or EM2-expressing Jurkat T-cells with Mtb-infected primary CD1c-expressing DCs would introduce a major confounder: reduced T-cell activation could reflect loss of CD1c expression rather than absence of a CD1c-dependent Mtb-induced activating signal. This is precisely why we used the engineered THP1-CD1c system, in which CD1c expression is preserved during Mtb infection (Fig. 2D). This model allowed us to test whether Mtb infection enhances CD1c-TCR-dependent activation without the confounding effect of infection-induced CD1c loss.

Using this controlled system, we show that EM1 and EM2 TCRs confer CD1c-endo tetramer binding, activation in response to plate-bound CD1c-endo protein, activation in response to THP1-CD1c but not THP1-KO APCs, and enhanced activation in response to Mtb-infected THP1-CD1c APCs (Fig. 5B-D). These data support the conclusion that the enhanced response to Mtb-infected APCs is mediated through CD1c recognition by the TCR.

We have revised the Results and Discussion to clarify this rationale and to explain why the engineered THP1-CD1c system was necessary for these experiments (revised manuscript, page 10, lines 242-244; page 17-18, lines 411-430).

(9) Conduct an experiment to assess T-cell cytotoxicity expressing EM1 or EM2, in the presence and absence of Mtb infection.

We agree this would be a valuable experiment. As outlined in our response to 8, EM1 and EM2 were cloned into Jurkat T-cells to test TCR-dependent CD1c recognition and activation, not cytotoxic effector function. Jurkat T-cells are not cytotoxic effector cells^3^, so they are not suitable for target-cell killing assays.

Cytotoxicity was instead assessed using the original CD1c-autoreactive T-cell lines (Figs. 3F-G and 4D-E). Testing EM1- or EM2-mediated killing would require engineering and validating primary human T-cells expressing these TCRs, which is a substantial additional workflow. We have clarified in the revised manuscript that the EM1/EM2 experiments demonstrate TCR-dependent recognition, while cytotoxicity was assessed using the CD1c-autoreactive T-cell lines (revised manuscript, page 10, lines 234–244).

(10) A list of primers used for TCR sequencing should be provided.

We have now provided the primer sequences used for targeted single-cell TCR sequencing in a new supplementary table (Table S1). We have also revised the Methods to provide additional detail on the single-cell TCR sequencing workflow, including CD1c-endo tetramer-guided single-cell sorting, oligo-dT reverse transcription, universal cDNA amplification, targeted amplification of TCR variable regions using TRAC-, TRBC-, TRGC- and TRDC-specific primers, well-specific 8-bp barcoding, size selection, library preparation and MiSeq sequencing. In addition, we now cite the SMART-seq2 protocol on which the approach was based (Picelli et al., 2014) (revised manuscript, page 20, lines 491-503; new Table S1).

**Reviewer #2 (Public review):**

Strengths:

(1) The study is designed well and has developed many exciting tools to generate specific information.

We thank the reviewer for this positive assessment of the study design and for recognising the value of the experimental tools developed in this work.

Weaknesses:

(2) The study has weaknesses in two important parameters - novelty and relevance in controlling TB. Further, the results could be better presented and discussed to allow easy understanding of the experimental design

We accept that the novelty and relevance to TB control could be made clearer in the manuscript. However, we believe the study makes several important and previously unreported contributions, and we have revised the Introduction, Results and Discussion to improve the clarity of the experimental design and to state the conceptual advance more explicitly.

First, to our knowledge, this is the first study to demonstrate that human CD1c-autoreactive T-cells respond more strongly to Mtb-infected CD1c+ APCs than to uninfected CD1c+ APCs. Previous work has shown that CD1c-autoreactive T-cells exist in human blood and can respond to CD1c-expressing cells in the absence of exogenous antigen. However, their role during infection has remained unclear. Our findings extend the field beyond the established steady-state, autoimmune and tumour contexts of CD1c autoreactivity by identifying Mtb-infected APCs as a biologically relevant setting in which these cells acquire enhanced effector activity. We propose that CD1cautoreactive T cells may not simply represent autoreactive bystanders that become pathogenic in disease, but instead form an evolutionarily conserved arm of lipid immune surveillance that can detect infection-associated changes in antigen presentation. Given the long-standing selective pressure imposed by microbial infection throughout human evolution, it is plausible that protection against infection represents a central physiological function of these cells, with their roles in autoimmunity and cancer reflecting the same capacity to sense altered self-lipid landscapes in other settings. Our data provide initial functional evidence supporting this model.

Second, the study provides functional evidence that these cells are not simply activated by infected APCs, but can mediate effector functions relevant to antimicrobial immunity. CD1c-autoreactive T-cells showed enhanced activation, cytokine production and cytotoxicity in response to Mtb-infected APCs, and led to reduced Mtb burden under in vitro conditions. These findings are directly relevant to TB immunity because cytotoxic T-cell pathways and antimicrobial molecules such as granulysin have been implicated in control of intracellular Mtb.

Third, the study links these functional observations to the ex vivo biology of human CD1c-autoreactive T-cells. Single-cell transcriptomic profiling demonstrates that these cells are enriched for cytotoxic effector-memory programmes and express molecules associated with target-cell killing and antimicrobial activity. This provides an independent, unbiased cellular basis for the functional assays and strengthens the conclusion that CD1c-autoreactive T-cells represent a plausible effector population in anti-mycobacterial immunity.

We agree that the experimental design needed clearer presentation. In the revised manuscript, we have improved signposting of the stepwise logic of the study: (1) defining and validating the THP1-CD1c APC system, (2) demonstrating CD1cautoreactive T-cell enrichment and specificity, (3) testing responses to UV-killed and live Mtb, (4) confirming TCR-dependent CD1c recognition using EM1 and EM2 TCR transfer, and (5) integrating these functional data with single-cell transcriptomic profiling of ex vivo CD1c-autoreactive T-cells. These revisions aim to make the experimental design easier to follow and to clarify how each section supports the overall conclusion.

We have revised the Introduction and Discussion accordingly to more clearly state the novelty and TB relevance of the work (revised manuscript, page 4-5, lines 83-104; page 14, lines 323-329).

(3) At several places, UV-killed or live Mtb were used. What is the rationale behind that?

We have now added a Methods statement explaining that UV-killed Mtb was used for controlled exposure to defined amounts of Mtb-derived antigen, particularly in dose-response cytotoxicity and cytokine-release assays, whereas live Mtb was used to assess T-cell activation, target-cell lysis and relative bacterial burden during APC infection with proliferating Mtb. We have also ensured that the figure legends clearly specify whether UV-killed or live Mtb was used in each experiment (revised manuscript, page 22, lines 534-540).

(4) Why use irradiated THP1-CD1c cells for activating T-cells?

Irradiated THP1-CD1c cells were used only during the T-cell expansion phase to provide sustained CD1c-mediated stimulation while preventing proliferation of the THP1 APCs. This was necessary because the expansion cultures lasted up to 12 days, during which non-irradiated THP1 cells would continue to divide and could overgrow the T-cell culture. Irradiation therefore allowed THP1-CD1c cells to function as APCs while maintaining controlled culture conditions and enabling selective expansion of CD1c-reactive T-cells. We have clarified this rationale in the Methods (revised manuscript, page 19-20, lines 472-474).

(5) While functional assays identified only CD4+ cells as CD1c-restricted, scRNAseq shows that both CD4+ and CD8+ cells exhibit this phenotype

We agree with the reviewer’s observation and have clarified this point in the revised Discussion. The functional assays were performed using CD1c-autoreactive Tcell lines generated from two donors. Both lines were CD4+αβTCR+, reflecting the outcome of the enrichment, sorting and expansion process used to generate sufficient T-cells for functional assays. These lines therefore provide mechanistic evidence that CD4+ CD1c-autoreactive T-cells can recognise CD1c+ APCs and respond more strongly to Mtb-infected APCs, but they are not intended to represent the full diversity of the CD1c-autoreactive T-cell compartment.

By contrast, the single-cell RNA-seq analysis was designed to provide a broader ex vivo assessment of CD1c-endo-binding T-cells without relying on prolonged in vitro expansion. This revealed that CD1c-autoreactive T-cells include both CD4+ and CD8+ populations, with enrichment of cytotoxic effector-memory programmes. We therefore interpret the functional and single-cell datasets as complementary: the functional assays provide mechanistic validation using tractable CD1c-reactive T-cell lines, while the single-cell data demonstrate that the broader ex vivo CD1c-autoreactive compartment is phenotypically diverse and includes both CD4+ and CD8+ cytotoxic populations.

We have revised the Discussion to clarify this point and to emphasise that combining in vitro functional assays with ex vivo single-cell profiling allowed us to capture both mechanistic activity and broader cellular diversity (revised manuscript, page 14-15, lines 341-349).

(6) Identifying the specific lipid antigen presented by CD1c could add greater value to the study.

We agree that identifying the specific CD1c-presented lipid antigen(s) would add important mechanistic insight. As noted in the response to the Editor, our data suggest that the enhanced response to Mtb-infected THP1-CD1c APCs is most likely due to altered CD1c-associated lipid presentation. However, defining these lipid species would require isolation of CD1c from infected APCs followed by specialised mass spectrometry-based lipidomics, which is a substantial additional workflow. We have revised the Discussion to state this limitation clearly and to highlight lipid identification as an important next step (revised manuscript, page 16-17, lines 380-410).

(7) Since autoreactivity was independent of exogenous antigen, the cytotoxic activity should also be independent of exogeneous antigens? What additional signal a THP1-CD1c cells treated with UV-killed Mtb express that is absent from the untreated cells?

CD1c-autoreactive T-cells likely recognise self-lipids presented by CD1c, but our data show that this response is enhanced after Mtb exposure. We interpret this as evidence that Mtb alters the quality or abundance of CD1c-associated lipid ligands, potentially through Mtb-derived lipids or infection-induced changes in host lipid metabolism. We have revised the Discussion to clarify that the precise lipid ligand(s) remain unidentified and will require future CD1c-lipidomic analysis (revised manuscript, page 16-17, lines 380-410).

(8) The relative Mtb growth assay is confusing. CD1c cells with Mtb infection triggers massive lytic response, as shown in Figure 4. Under similar conditions, in Figure 6, the authors report a significant decline in Mtb growth in these cells. The problem is that with the kind of lytic response observed, a lot more Mtb could be present extracellularly and would evade killing. How do we reconcile the two observations?

In the Mtb lux assay, extracellular bacteria were removed by washing after the initial infection step, so the starting bacterial population measured in the co-culture assay is expected to be predominantly cell-associated. We also recognise that the luminescence readout measures total viable lux-expressing Mtb under the assay conditions and does not distinguish intracellular from extracellular bacteria at later time points.

The cytotoxicity observed in Fig. 4 reflects enhanced but incomplete lysis of infected APCs. Therefore, although T-cell-mediated lysis could release some bacteria from infected target T-cells, the reduced luminescence observed in Fig. 6C indicates a lower net viable Mtb burden under these co-culture conditions. We interpret this as the combined outcome of CD1c-autoreactive T-cell effector activity, including cytotoxicity and antimicrobial mediators such as granulysin and cytokines, rather than as direct evidence of selective intracellular bacterial killing.

We have revised the Results and Methods to clarify that the Mtb lux assay measures relative viable bacterial burden/luminescence under in vitro co-culture conditions. We have changed the discussion to avoid over-interpreting this assay as distinguishing intracellular from extracellular Mtb killing (revised manuscript, page 11-12, lines 266274, page 26, lines 633-637).

**Reviewer #2 (Recommendations for the authors):**

(9) Nearly 40-50% of the samples did not respond to THP1-CD1 stimulation. What contributes to this diversity?

We agree that there is clear donor-to-donor variability in the response to THP1-CD1c stimulation [4]. Approximately one-third of donors did not show detectable expansion under these assay conditions. This likely reflects differences in the precursor frequency and TCR repertoire composition of CD1c-autoreactive T-cells between donors, together with variation in activation state and responsiveness during short-term in vitro expansion. Apparent non-response may also reflect low-frequency CD1c-reactive populations that are present but fall below the detection threshold. We have revised the Discussion to acknowledge donor heterogeneity as an expected feature of primary human CD1c-autoreactive T-cell responses (revised manuscript, page 14-15, lines 341-349).

(10) For lung biopsy staining, how is CD1 expression in healthy tissue or some unrelated inflammatory condition?

The purpose of the lung biopsy staining was to determine whether CD1c-expressing cells are present in human TB lung tissue and to assess their spatial relationship to granulomatous inflammation, rather than to perform a formal comparison between healthy, non-TB inflammatory and TB lung tissue.

Across the TB biopsies analysed, CD1c staining was spatially heterogeneous. CD1c expression was generally low or infrequent in central granuloma regions and in tissue regions remote from granulomatous inflammation, whereas staining was more apparent in distal inflammatory tissue and lymphoid/B-cell follicle-rich regions. We have revised the Results to clarify that these data are presented as representative spatial observations within TB lung tissue, rather than as a quantitative comparison with healthy or unrelated inflammatory tissue.

We agree that comparison with healthy lung and non-TB inflammatory lung tissue would provide useful additional context, particularly for distinguishing TB-associated changes from more general inflammatory induction of CD1c. We now acknowledge this as an important future direction (revised manuscript, page 18, lines 425-430). We have also revised the Results to clarify the spatial pattern of CD1c staining within TB lung tissue (revised manuscript, page 7, lines 144-151).

(11) What was the rationale for using UV-killed or live Mtb for different experiments?

This point is addressed in our response to 3 above.

**Reviewer #3 (Public review):**

Strengths:

(1) The manuscript is well written, and the novelty, impact, and limitations of this study are precisely highlighted by the authors.

We thank the reviewer for this positive assessment of the manuscript, particularly their recognition of the study’s novelty, impact and balanced discussion of its limitations.

(2) Lipid antigen identification and direct lipid identification via lipidomics/MS of CD1c-bound lipids from Mtb-infected APCs would clarify whether the enhancement arises from altered self-lipids or subtle Mtb lipids

We agree that direct identification of CD1c-bound lipids from Mtb-infected APCs would provide important mechanistic insight and help determine whether enhanced activation reflects altered self-lipids, Mtb-derived lipids, or shared lipid species. As noted above, this would require isolation of CD1c from infected APCs followed by specialised mass spectrometry-based lipidomic analysis, which represents a substantial additional workflow. We have revised the Discussion to state this limitation clearly and to highlight CD1c-lipidomic analysis as an important next step (revised manuscript, page 16-17, lines 380-410).

**Reviewer #3 (Recommendations for the authors):**

(3) Figure 2Ai-vi, lines 134-136. The authors should include the data from central granuloma staining to solidify their claim of the presence of CD1c expression remote from the centre of TB granulomas.

Central granuloma regions are included in Fig. 2A, including panels showing staining within granulomatous tissue where CD1c expression is low or infrequent compared with distal inflammatory and lymphoid/B-cell follicle-rich regions. We agree that this spatial distinction was not sufficiently clear in the original text and figure legend.

We have therefore revised the Results and Fig. 2 legend to more explicitly guide the reader through the central versus distal regions shown in Fig. 2A. The revised text now states that CD1c expression was observed across lung biopsies from all five TB patients, but was spatially heterogeneous, with staining most apparent in distal inflammatory tissue and lymphoid/B-cell follicle-rich areas, and generally low or infrequent in central granuloma regions (revised manuscript, page 7, lines 144-151; Fig. 2 legend).

(4) Figure 2D, lines 149-151. The authors should clarify whether the CD1c resistance to downregulation is model-specific to THP1-CD1c-APCs or an overexpression artefact

As described in our response to Reviewer 1, 5, we agree that this point required clearer explanation. We have clarified in the revised Results and Discussion that the preservation of CD1c expression in THP1-CD1c APCs likely reflects the engineered nature of this system, rather than a general feature of endogenous CD1c regulation during Mtb infection.

Specifically, in primary MoDCs, Mtb infection reduces CD1c expression at both transcript and protein levels (Fig. 2B and 2C). In contrast, CD1c in THP1-CD1c APCs is expressed from a heterologous CD1c-β2m fusion construct rather than from the endogenous CD1C locus. Therefore, its resistance to downregulation is likely model-specific and related to the expression system. We now state this in the Results and Discussion, and explain that this feature allows CD1c-dependent T-cell responses to Mtb-infected APCs to be assessed without the confounding effect of infection-induced CD1c loss (revised manuscript, page 7-8, lines 165-174; page 17-18, lines 411-430).

(5) Figure 6C. The relative Mtb burden is measured through luminescence. While this correlates closely with CFUs, confirmation with plating is better evidence.

We have previously shown close correlation between luminescence in our system and CFUs^5^ (Bielecka mBio 2017, PMID: 28174307). Perhaps controversially, we propose that luminescence is a better readout of total Mtb load. Luminescence captures all metabolically active Mtb, whilst CFUs may be confounded by clumping of bacteria, for example, giving an underestimate. However, we agree with the reviewer that luminescence is an indirect measure of bacterial burden and that CFU plating would provide additional confirmatory evidence. We have therefore revised the Results, Discussion and Methods to describe the assay more cautiously as a measure of relative viable Mtb burden/luminescence, and we now acknowledge the absence of CFU confirmation as a limitation of the study (revised manuscript, page 11-12, lines 266-274; page 17, lines 400-403; page 26, lines 633-637).

(6) Figure 7. The authors show that CD1c-autoreactive T-cells exhibit cytotoxic effector memory phenotype. While the sc-RNAseq subsampling is robust, the number of sample donors being 2 might create a potential bias.

We agree that the use of two donors for the single-cell RNA-seq analysis is a limitation and could introduce donor-specific bias. We have now stated this more explicitly in the Discussion. Importantly, the scRNA-seq data are not used alone to define function, but rather provide an ex vivo phenotypic framework that complements the functional assays showing CD1c-dependent activation, cytokine production, cytotoxicity and reduced relative Mtb burden.

The subsampling analysis supports the robustness of the transcriptional patterns within this dataset, but we agree that larger donor cohorts will be required to determine how consistently these cytotoxic effector-memory programmes are represented across the broader human CD1c-autoreactive T-cell compartment. We have revised the Discussion accordingly (revised manuscript, page 17, lines 403-410).

(7) The authors should on how it might compare with non-autoreactive CD1c-restricted T-cells.

We agree that it is important to place these findings in the context of non-autoreactive, antigen-specific CD1c-restricted T-cells. Previous studies have shown that CD1c can present microbial lipid antigens, such as mycobacterial lipids, to T-cells with defined antigen specificity. In contrast, the CD1c-autoreactive T-cells studied here recognise endogenous ligands and appear to respond to infection through changes in the CD1c-presented lipid repertoire rather than through recognition of a single defined foreign antigen.

Our findings suggest that autoreactive CD1c-restricted T-cells may provide a complementary mode of immune surveillance, capable of sensing infection-induced changes in lipid presentation, whereas non-autoreactive CD1c-restricted T-cells may respond more directly to specific microbial lipid antigens. A head-to-head comparison would clearly be very interesting, but an extensive new piece of work beyond the scope to the current study. We have expanded the Discussion to more clearly highlight this distinction, and that direct comparison is required (revised manuscript, page 16-17, lines 380-410).

Concluding remarks

In summary, we have addressed the key concerns raised by the reviewers by strengthening validation of the experimental system, improving characterisation of T cell populations, and clarifying mechanistic interpretation. We believe these revisions significantly improve the clarity and rigour of the manuscript. We accept that identification of the CD1-presented lipids is an important next step that will give significant mechanistic insight, but is beyond the scope of the current work.

References:

(1) Wen, Q. et al. MiR-381-3p Regulates the Antigen-Presenting Capability of Dendritic Cells and Represses Antituberculosis Cellular Immune Responses by Targeting CD1c. J Immunol 197, 580-589 (2016). https://doi.org/10.4049/jimmunol.1500481

(2) Gagliardi, M. C. et al. Bacillus Calmette-Guerin shares with virulent *Mycobacterium tuberculosis* the capacity to subvert monocyte differentiation into dendritic cell: implications for its efficacy as a vaccine preventing tuberculosis. Vaccine 22, 3848-3857 (2004). https://doi.org/10.1016/j.vaccine.2004.07.009

(3) Grailer, J. et al. A Novel Cell-based Luciferase Reporter Platform for the Development and Characterization of T-Cell Redirecting Therapies and Vaccine Development. J Immunother 46, 96-106 (2023). https://doi.org/10.1097/CJI.0000000000000453

(4) Guo, T. et al. A Subset of Human Autoreactive CD1c-Restricted T Cells Preferentially Expresses TRBV4-1(+) TCRs. J Immunol 200, 500-511 (2018). https://doi.org/10.4049/jimmunol.1700677

(5) Bielecka, M. K. et al. A Bioengineered Three-Dimensional Cell Culture Platform Integrated with Microfluidics To Address Antimicrobial Resistance in Tuberculosis. mBio 8 (2017). https://doi.org/10.1128/mBio.02073-16
